# Supercritical CO_2_ treatment reduces the antigenicity of buttermilk β-lactoglobulin and its inflammatory response in Caco-2 cells

**DOI:** 10.3168/jdsc.2020-0028

**Published:** 2020-12-11

**Authors:** Israel García-Cano, Po-Wei Yeh, Diana Rocha-Mendoza, Rafael Jiménez-Flores

**Affiliations:** Department of Food Science and Technology, Parker Food Science & Technology Building, The Ohio State University, Columbus 43210

## Abstract

β-Lactoglobulin (β-LG) is believed to be a common allergen in bovine milk. Buttermilk (BM) powder has abundant contents of milk fat globule membrane and phospholipid, both of which have been demonstrated to have positive effects on brain and cognitive development during early infancy. This study focused on modifying β-LG in BM via supercritical CO_2_ (ScCO_2_) treatment to modify its reactivity to antibodies and thus reduce its antigenicity. Buttermilk powder was treated in a supercritical fluid extraction system with food-grade CO_2_ at 100, 150, 200, 250, 350, and 400 bar at 2 temperatures, 50 and 75°C. All analyses were completed in a 10% BM suspension (wt/vol). The BM proteins were examined using sodium dodecyl sulfate (SDS)-PAGE, Western blot, ELISA, and periodic acid staining methods. Semi-purified β-LG was used to evaluate the cytotoxicity, viability, and inflammatory response in the Caco-2 cell line by means of the lactate dehydrogenase assay, MTT [3-(4,5-dimethylthiazol-2-yl)-2,5-diphenyltetrazolium] assay, and IL-8 production, respectively. The SDS-PAGE showed that the signal intensity of β-LG bands was reduced by up to 50% after being processed at 250 bar and 75°C for 30 min. Lighter and more diffuse signals were found by Western blot, indicating modification of the protein structure. The ELISA demonstrated that ScCO_2_ treatment could significantly change β-LG antigenicity in BM. Sugar moieties in bands corresponding to β-LG were revealed by periodic acid staining, indicating glycosylation only in samples treated with ScCO_2_. Caco-2 cells treated with whey proteins had high viability, 24.9% lower inflammation, and no evidence of cytotoxicity compared with untreated cultures. These results showed that reduced antigenicity of β-LG was caused by lactosylation, which has been reported as a possible pathway to reduce the allergenicity in foods. The denaturation of β-LG by supercritical fluid processing is a promising way to address milk allergy, which remains a problem requiring more attention and further research.

## Highlights

• This paper shows the use of ScCO_2_ and the different functionality in milk proteins
• Data accumulation of ScCO_2_ application goes beyond current industrial applications
• ScCO_2_ process can improve nutritional or physiological characteristics of proteins
• Exposing proteins to ScCO_2_ can induce changes in their structure and function
• The new generation of food scientists welcomes a process with low environmental impact

## Graphical Abstract

**Figure:**
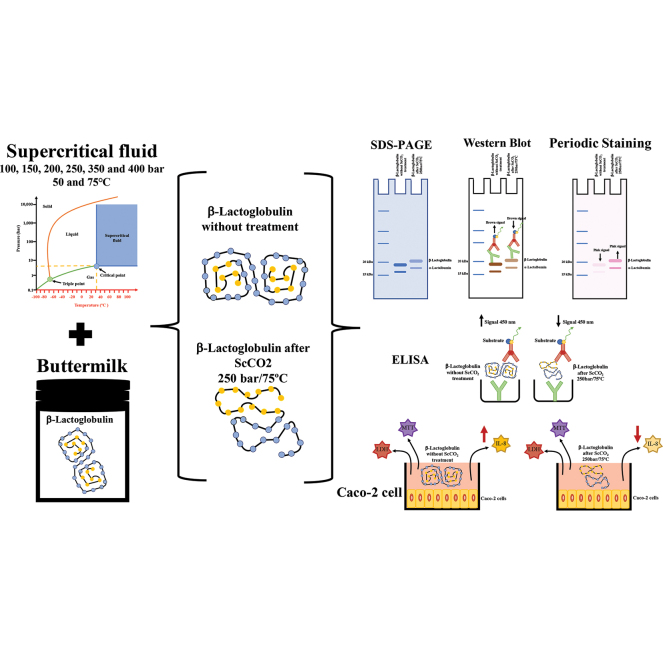
**Summary:** We present an original approach to treatment of buttermilk with supercritical (Sc)CO_2_ with the aim of modifying protein functionality. We used the ScCO_2_ chamber with minimal flow of supercritical fluid (mimicking a reaction chamber) and treatment variations in temperature/pressure exposure. Results show that modifications were directed only on β-LG, mainly at 250 bar and 75°C, resulting in changes in mobility and its interaction with antibodies and response from Caco-2 cells. The sensitivity of the antibody to the modified β-LG was diminished significantly, and although the response of Caco-2 cells on viability and toxicity did not differ across treatments, we detected a significant reduction in IL-8 response that can be interpreted as reduction of the inflammatory response.

The most common food allergens are contained in 8 foods: cow milk, fish, crustaceans and shellfish, eggs, soy, peanuts, nuts, and wheat, which together account for more than 90% of all serious allergic reactions to food worldwide (Bu et al., 2013). Bovine milk allergy is found in 2 to 6% of infants and is considered the most prevalent food allergy. Caseins, β-LG, and α-LA are the main allergens in cow milk (Golkar et al., 2019). Therefore, it is important to find new and effective processing technologies to reduce the allergen response in humans to cow milk as potential means to control milk allergy. Buttermilk (**BM**) powder has abundant milk fat globule membrane and phospholipid contents, both of which have been demonstrated to have benefits in brain health and cognitive development during early infancy (Fontecha et al., 2020). Supercritical CO_2_ (**ScCO_2_**) treatment has been applied to extract phospholipids from BM using ethanol as a co-solvent. This phospholipid-rich extract has a potential application in functional foods (Sprick et al., 2019; Ubeyitogullari and Rizvi, 2020).

Thermal treatment and hydrolysis are the most common processing methods used to reduce allergenicity of food proteins. Nevertheless, treatment with ScCO_2_ has generated interest because of its application in the modification of polymers (Xu et al., 2011). Supercritical CO_2_ has been used in functional dairy beverages without causing differences in physicochemical properties or color (Silva et al., 2019). Additionally, the ScCO_2_ process offers many advantages in preserving food quality by inactivating microorganisms and enzymes in liquid foods (Xu et al., 2011).

Supercritical CO_2_ has been shown to be a feasible processing method for modifying milk proteins; Amaral et al. (2017), Striolo et al. (2003), and Xu et al. (2011) showed that whey proteins had different functionalities after their structure and conformation were modified by ScCO_2_. The change in secondary structure of whey protein ingredients treated with ScCO_2_ revealed a decrease in α-helix structures and hydrogen bonds and an increase in β-sheet structures (Xu et al., 2011). In addition, conjugation of proteins with reducing sugars via the Maillard reaction, which can occur under ScCO_2_ treatment, is an effective means of improving the functional properties of proteins. Conjugation of β-LG with carboxymethyl dextran improves emulsifying properties, increases thermal stability, and reduces the immunogenicity of this protein (Inada et al., 2015). Inada et al. (2015) found that β-LG conjugated with soybean soluble polysaccharide improved emulsifying properties and reduced immunogenicity tested in mice.

The aim of this study was to modify β-LG from BM powder to reduce its antigenicity using ScCO_2_ processing. One hundred grams of BM powder (Dairy America, Fresno, CA) was treated using ScCO_2_. The sample was placed in a 1-L stainless-steel extraction vessel equipped with a heat jacket for 30 min of preheating at the assigned temperatures. The supercritical fluid extraction system and ChromScope IE operating program were from Waters (Milford, MA). The conditions used in the extraction vessel were 100, 150, 200, 250, 350, and 400 bar. Treatment temperature was controlled at 50 and 75°C for each pressure condition. Each batch treatment cycle was as follows: (1) start: dynamic flow of ScCO_2_, (2) static or “soaking,” (3) dynamic flow, (4) static, and (5) dynamic flow. For each cycle, a 10-min operating time was set for the first 3 durations (dynamic → static → dynamic); then, the sample was treated for 30 min in step 4 (static) and for 20 min in step 5 (dynamic). Food-grade ScCO_2_ flowed at a rate of 100 g/min in the dynamic steps. After the ScCO_2_ treatment of each BM sample, powder samples were resuspended in water to obtain a 10% total solids suspension. The samples were rehydrated under gentle agitation at 25°C for 1 h and then incubated at 4°C overnight for complete hydration. The suspensions were centrifuged (Sorvall Legend XT/XF Centrifuge, ThermoFisher Scientific, Waltham, MA) at 2,700 × *g* for 30 min, and the supernatant was collected for future experiments. Thermal treatment-only control samples were incubated at 50 and 75°C for 80 min; however, we decided to use only the control sample treated at 75°C because there were no significant differences between the 50°C control treatment and no treatment. Total soluble protein was quantified by micro-bicinchoninic acid (BCA) method, following the manufacturer's instructions (Protein Assay kit; ThermoFisher Scientific). The BM samples were analyzed by SDS-PAGE using 4–20% stain-free precast gels (Bio-Rad Laboratories, Hercules, CA). Equal amounts of total soluble proteins from each sample were loaded. The running conditions were 20 min at 90 V and 40 min at 160 V. The gel was analyzed by densitometry using ChemiDoc Touch Imaging System (Bio-Rad Laboratories). To standardize the quantification, we used the proportional relationship between band intensity and sample concentration; thus, the amount of β-LG in each lane could be determined with reference to a standard curve. The edges of a peak were defined as the intensity of approximately 0.01% of selected area volume.

After SDS-PAGE analysis, Western blot, ELISA, and periodic acid staining were performed. The samples were fractionated using Amicon Ultra Centrifugal Filters (Merck Millipore, Burlington, MA) with membrane pore sizes of 100, 30, and 10 kDa cut-off. Western blotting against β-LG was performed using the Trans-Blot Turbo Transfer System (Bio-Rad Laboratories) using a Trans-Blot Turbo Mini PVDF Transfer Packs onto 0.2-mm polyvinylidene difluoride (PVDF) membranes (Bio-Rad Laboratories), following the manufacturer's instructions. Primary antibody (rabbit anti-β-LG; 1:20,000) and secondary antibody (horseradish peroxidase–conjugated goat anti-rabbit antibody; 1:10,000) were purchased from GeneTex (Irvine, CA). The brown signals on PVDF membranes were revealed by immersing in 3,3′-diaminobenzidine solution prepared from the DAB kit (Sigma-Aldrich, St. Louis, MO) for 10 min. The ELISA was performed using a bovine β-lactoglobulin ELISA Quantitation Kit (Bethyl Laboratories Inc., Montgomery, TX) according to the manufacturer's instructions. Absorbance was measured using a plate reader spectrophotometer (accuSkan Go UV-Vis; ThermoFisher Scientific) at 450 nm. Periodic acid staining was performed to detect possible glycosylation. The SDS-PAGE gel with semi-purified proteins was stained by Pierce Glycoprotein Staining Kit (ThermoFisher Scientific). All reagents were prepared and experiments were carried out according to the manufacturer's instructions. Horseradish peroxidase was used as a positive control. The purified proteins were tested at concentrations of 1 mg/mL in in vitro experiments using the intestinal Caco-2 cell line. Cell viability, using the 3-(4,5-dimethyldiazol-2-yl)-2,5 diphenyl tetrazolium bromide (MTT) assay (Millipore, Billerica, MA), and cytotoxicity, using the lactate dehydrogenase assay (Roche, Mannheim, Germany),were performed according to Rocha-Mendoza et al. (2020). The concentration of IL-8 was determined using a human IL-8 CXCL8 ELISA Assay Kit (Sigma-Aldrich) following the manufacturer's instructions. The plate was read at 450 nm using a plate reader (accuSkan Go UV-Vis spectrophotometer). As a positive control of inflammation, Caco-2 cells were stimulated with a mix of 25 ng/mL IL-1 and 10 μg/mL LPS from *Escherichia coli* (Sigma-Aldrich). The results were obtained by taking the mean of the 4 experimental independent experiments and 3 replicates.

Statistical analyses were accomplished using SPSS software (version 23; IBM Corp., Armonk, NY). One-sample *t*-test was used for the comparison between experimental data of ScCO_2_-treated samples and controls; *P* < 0.05 was considered to indicate significant results.

The BCA results confirmed that the same amount of soluble protein in each processed BM powder was loaded on the SDS-PAGE gel. Figure 1A shows the protein profile after different ScCO_2_ pressures at 50 and 75°C. In Figure 1, the whey proteins (β-LG and α-LA) moved up and showed fuzzy bands after treatment with ScCO_2_ at 75°C with pressure >250 bar. The bands increased their molecular weight by around 22% (4 kDa). These variations suggest posttranslational modifications on whey proteins that caused an increase of molecular mass. These modifications have been reported by Morgan et al. (1997), where nonenzymatic lactosylation of β-LG and α-LA can occur with some sugar fragments during ScCO_2_ treatment. Sugar attachment has been reported to reduce the immunogenicity of β-LG (Corzo-Martínez et al., 2010; Milkovska-Stamenova and Hoffmann, 2016; Xu et al., 2018). Fuzzy whey protein bands were not observed in any sample treated at 50°C, including the control, demonstrating that temperature affected the modification. However, temperature was not the only factor leading to modifications. The β-LG band of the heat control incubated in 75°C and at atmospheric pressure did not display any variation in molecular mass, which was further evidence that ScCO_2_ had a denaturing effect on β-LG different from thermal processing or pressurization. Moreover, band variations only occurred in whey proteins; caseins were not influenced by the modification. Figure 1B shows the amount of β-LG contained in processed BM and quantified using Image Lab software. The β-LG concentration was significantly reduced (*P* < 0.05) when BM powder was treated with ScCO_2_ at 75°C at pressures ranging from 100 to 400 bar. The β-LG content declined from 3.39 ± 0.28 mg/mL in the original BM to 1.69 ± 0.13 mg/mL in the sample treated with ScCO_2_ at 250 bar and 75°C, a reduction of approximately 50%. As the most abundant sugar in dairy products, lactose (or lactose residues) potentially reacts with proteins to form glycoproteins under specific conditions (Fogliano et al., 1998). Therefore, the observed decrease in β-LG content might be due to the attachment of glycan residues under the conditions evaluated.

**Figure 1:**
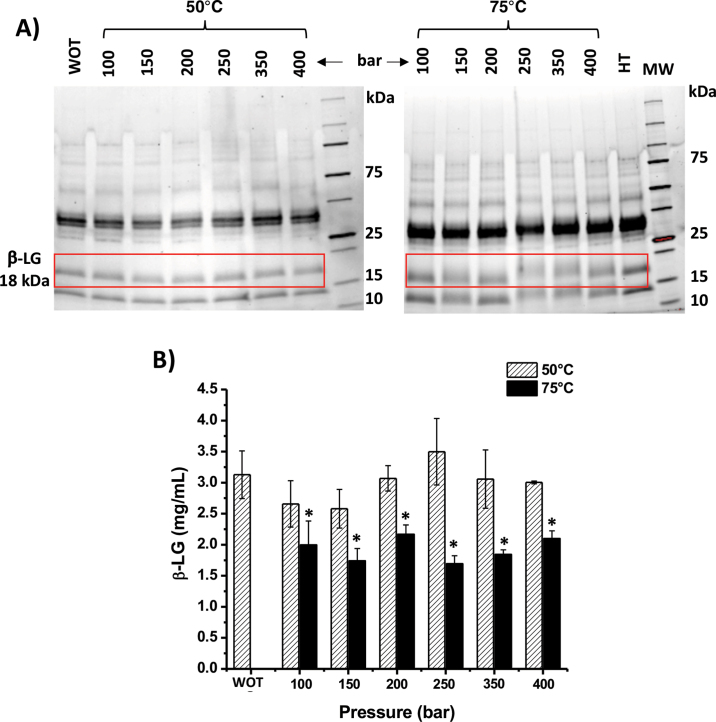
Buttermilk (BM) protein profile and β-LG quantification after different supercritical CO_2_ (ScCO_2_) conditions. (A) Protein profile of BM treated at 50 and 75°C under ScCO_2_ at different pressures (100 to 400 bar); the red box indicates the β-LG. WOT = without treatment; HT = heat treatment (75°C and no ScCO_2_); MW = molecular weight marker (All Blue, Bio-Rad Laboratories, Hercules, CA). (B) Quantification β-LG from BM treated at 50 and 75°C and under ScCO_2_ at different pressures. Error bars indicate mean ± SD of 3 independent experiments. Asterisks (*) represent significant differences between the original sample and treated samples (*P* < 0.05).

Quantitative results of β-LG antigenicity from BM using ELISA are shown in Figure 2A. More than half of the treatments significantly lowered (*P* < 0.05) β-LG antigenicity compared with the original BM. The antigenicity of β-LG decreased in treatments at 100 and 150 bar and 50°C (reductions of 25.2 and 42.2%, respectively). At 75°C and 250, 350, and 400 bar, the β-LG signal intensity decreased from 38.81 ± 0.49 ng/mL in the original BM to 22.93 ± 0.49, 24.93 ± 0.49, and 24.05 ± 0.36 ng/mL, respectively, representing reductions of 40.9, 35.7, and 38.0%, respectively, compared with the original BM. Figure 2B shows the Western blot, with wider bands in treatments of 250, 350, and 400 bar at 75°C; these wider bands show a range of molecular weights due to the covalent addition of lactose (results not shown from HPLC/MS analysis). This result contrasts with the reduced antigenicity in some bands treated at 50°C and 100 or 150 bar. These findings implied that the antigenicity of β-LG was reduced in the fuzzy bands due to change in conformation caused by lactosylation. After purification, 2 selected samples (without treatment and BM treated with ScCO_2_ at 250 bar/75°C) were loaded onto an SDS-PAGE gel and the gel was fixed, oxidized, and stained. Figure 2C shows a positive signal as evidence that the whey proteins were attached to some sugars during ScCO_2_ processing. The staining also displayed positive signals for α-LA. A weak signal was observed in the BM without treatment, which could be regarded as an effect of spray drying. In spray drying, evaporation of the water is caused by the heat exchange between the dry air stream and milk particles (Verhoeckx et al., 2015). Maillard reactions have been reported to be triggered during spray drying due to the temperature gradient of milk particles, and allergenicity can therefore be affected by the attachment of sugar fragments (Schuck et al., 2013).

**Figure 2:**
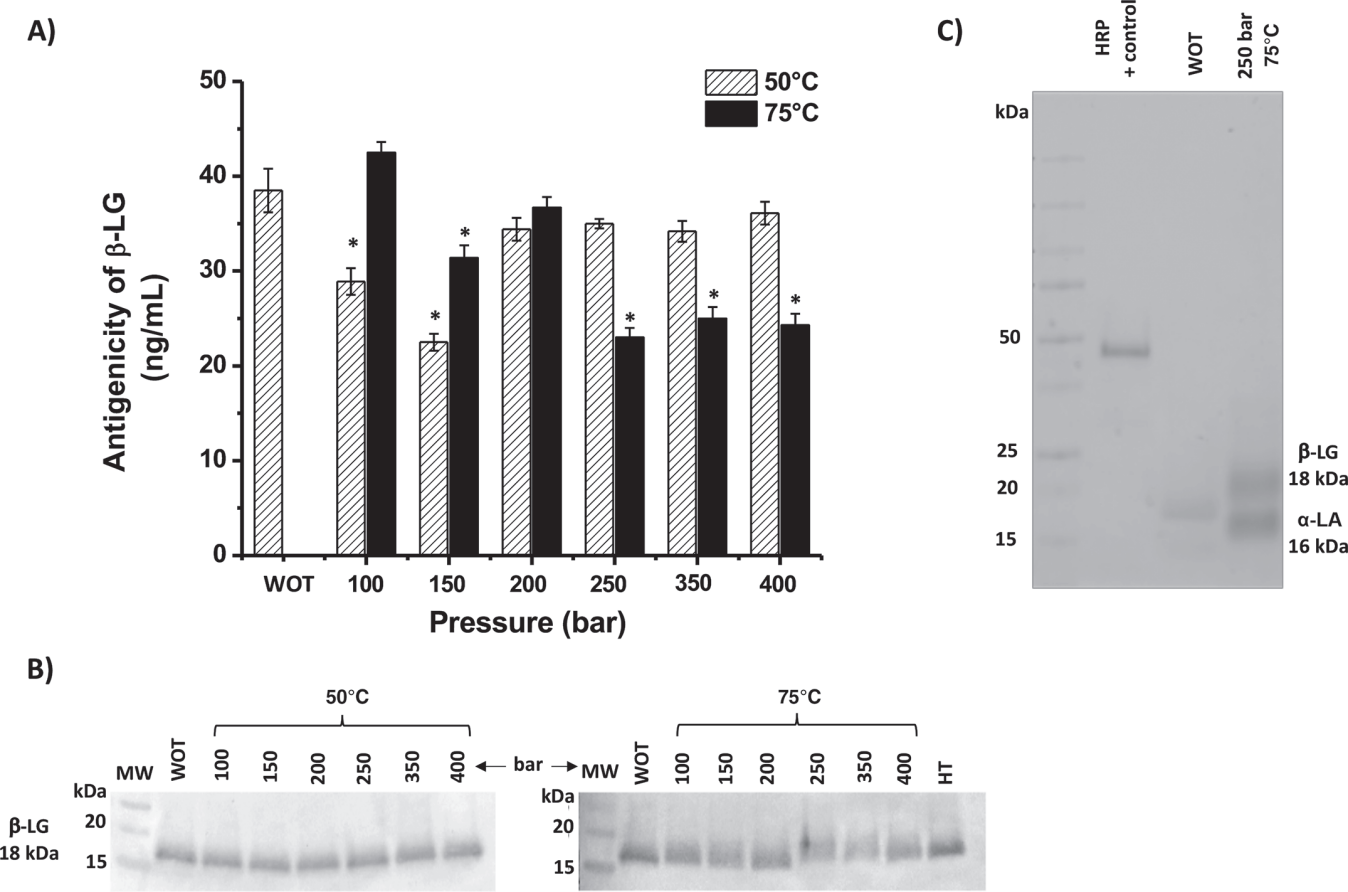
(A) Antigenicity of β-LG from buttermilk (BM) under different supercritical CO_2_ (ScCO_2_) conditions at 50 and 75°C tested by ELISA (results in ng/mL). The asterisk (*) represents significant differences between the original sample and experimental treatments (*P* < 0.05). Error bars indicate mean ± SD of 3 independent experiments. (B) Western blot against β-LG in BM treated at 50°C (left) and 75°C (right) and different pressures (100 to 400 bar). WOT = without treatment; HT = heat treatment (75°C and no ScCO_2_); MW = molecular weight marker. (C) Periodic acid staining of the BM sample treated at 250 bar and 75°C after semi-purification. Horseradish peroxidase (HRP) was used as a positive control.

We conducted an in vitro study using Caco-2 cells to assess the effect of ScCO_2_-treated proteins on cell viability, cytotoxicity, and inflammatory response of human epithelial cells. The mechanism of how milk and dairy products affect immune responses in the body is still unclear. Cytokines are thought to be important substances in inflammatory pathways because they are responsible for cell-to-cell communications (Ustunol and Wong, 2010). Interleukin-8 is a cytokine secreted by intestinal epithelial cells and regulated by food-derived substances. We selected it for monitoring in this study because it is a powerful chemoattractant and neutrophil activator that can attract monocytes and T cells to inflammatory sites, leading to mucosal inflammation as a side effect of an abnormal immunological response (Desjeux and Heyman, 1994). Accordingly, IL-8 is highly related to the initiation of the mucosal inflammatory response to an antigen and therefore affects antibody-forming cells and neutrophils, respectively (Satsu et al., 2004; Ustunol and Wong, 2010). Figure 3A shows the viability of Caco-2 cell line [using the MTT assay; 3-(4,5-dimethylthiazol-2-yl)-2,5-diphenyltetrazolium bromide, tetrazolium reduction]. After exposed to different BM proteins, Caco-2 cells maintained greater than 100% cell viability (versus PBS control as the baseline). Figure 3B shows the results of the cytotoxicity test using lactate dehydrogenase (LDH) detection; no treatments resulted in cellular cytotoxicity in excess of 5%. Hence, these results indicate that BM proteins would not damage cells during incubation. These assays and results for cytotoxicity and viability are important prerequisites for evaluating the effect of samples on cells; otherwise, the immune response of the cells could not be attributed to the tested substances. Figure 3C demonstrates IL-8 production by the Caco-2 cell line in the BM sample without and with heat treatment. Neither sample showed significant differences in IL-8 production. However, IL-8 production decreased 24.9% in cells exposed to β-LG from BM treated at 250 bar and 75°C. Because glycation of antigens has been reported to be an efficient way to inhibit IL-8 secretion in Caco-2 cells (Teodorowicz et al., 2013) and Maillard reaction products were found to have IL-8 inhibitory activities (Kitts et al., 2012), the result from the semi-purified sample could be used to demonstrate that lactosylation resulting from ScCO_2_ treatment at 250 bar and 75°C would reduce the inflammatory effects of β-LG and inhibit IL-8–stimulating reactions.

**Figure 3:**
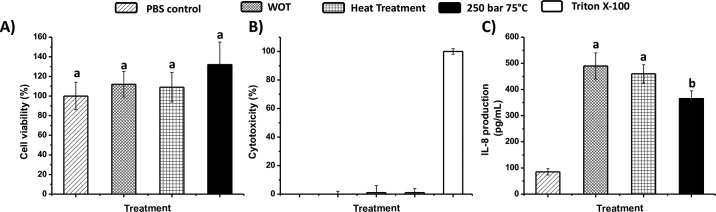
Determination of (A) cell viability (by MTT assay); (B) cytotoxicity (by lactate dehydrogenase assay); and (C) inflammation (IL-8 production) in Caco-2 cells, using semi-purified β-LG from buttermilk powder (BM) without and with treatment with supercritical CO_2_ (ScCO_2_) at 250 bar and 75°C. WOT = purified proteins from BM without treatment. Triton X-100 was from Sigma-Aldrich (St. Louis, MO). Error bars indicate mean ± SD of 4 independent experiments and 3 replicates. Different lowercase letters (a, b) indicate significance differences (*P* < 0.05).

In general, our results suggested that β-LG has a unique denaturing pattern in the presence of ScCO_2_, leading to conformational changes and an epitope-blocking effect achieved by lactosylation that could reduce the capacity to bind IgG antibodies. Our in vitro experiments demonstrated that IL-8 production in the Caco-2 cell line was decreased when cells were incubated with ScCO_2_-treated whey proteins compared with the original BM-sourced whey proteins. The decrease in IL-8 production indicated that the ScCO_2_ treatment could inhibit allergic reactions because an inflammatory response is the initial step of food hypersensitivity.

In summary, we demonstrated the feasibility of using our system with stagnant ScCO_2_, which makes the system a reaction vessel, where different reactions were induced in the structure of proteins, including an accelerated reaction with lactose or other hydrophobic molecules. We also observed that the reaction is specific for β-LG and that the changes induced reduce its antigenicity, which is at the base of allergic reactions.
